# Supplementary material for: Healthcare System Resilience and Adaptation: A Six-Year Analysis of Heart Failure Care in the Veterans Affairs System
Source: medRxiv. 2025 Oct 15:2025.10.13.25337954. Preprint. [Version 1] doi: 10.1101/2025.10.13.25337954 (PMC12633086; doi:10.1101/2025.10.13.25337954)
Supplement: 1 [file NIHPP2025.10.13.25337954V1-supplement-1.pdf]

medRxiv preprint doi: https://doi.org/10.1101/2025.10.13.25337954; this version posted October 15, 2025. The copyright holder for this preprint
(which was not certified by peer review) is the author/funder, who has granted medRxiv a license to display the preprint in perpetuity.
It is made available under a CC-BY 4.0 International license .

Supplemental Material

medRxiv preprint doi: https://doi.org/10.1101/2025.10.13.25337954; this version posted October 15, 2025. The copyright holder for this preprint (which was not certified by peer review) is the author/funder, who has granted medRxiv a license to display the preprint in perpetuity.
It is made available under a CC-BY 4.0 International license .

Supplemental Methods

For the survival analysis, the most recent clinical variable values in the year prior to 1/1/2019 were used. Race-stratified multiple imputation using chained equations across 5 datasets was performed to address missing data in clinical variables (BMI, heart rate, blood pressure, hemoglobin, eGFR, and LVEF), consistent with established guidance for large samples.[18,19] Nelson-Aalen cumulative hazard estimators were included in the imputation model to maintain survival structure and ensure missing variables were imputed conditional on observed survival patterns within each race/ethnicity.[45] Imputation adequacy was assessed by consistency of variable distributions across imputed datasets within each racial stratum as well as confirmation of clinical plausibility of all imputed values. Complete cases were defined as patients with no missing values in key covariates required for Cox regression models after creation of Nelson-Aalen cumulative hazard estimators. Kaplan-Meier survival curves were generated for visualization.

medRxiv preprint doi: https://doi.org/10.1101/2025.10.13.25337954; this version posted October 15, 2025. The copyright holder for this preprint (which was not certified by peer review) is the author/funder, who has granted medRxiv a license to display the preprint in perpetuity.
It is made available under a CC-BY 4.0 International license .

Table S1: Sensitivity Analysis: Seasonal vs Non-seasonal Models for All Outcomes

Table S1A: Model Diagnostics of Seasonal vs Non-seasonal Models for Medication Use

Medication	Model	R^2	R^2 adj	aic	bic
**ACE ARB ARNI**	Base	0.9861	0.9846	16.4821	28.1896
	Seasonal	**0.9945**	**0.9920**	**-9.7096**	**23.4615**
**ARNI**	Base	0.9976	0.9974	**95.1355**	**106.8430**
	Seasonal	**0.9984**	**0.9977**	96.5899	129.7610
**Beta-Blocker**	Base	0.8304	**0.8120**	**72.8511**	**84.5586**
	Seasonal	**0.8526**	0.7852	87.5550	120.7261
**MRA**	Base	0.9958	**0.9953**	**47.7982**	**59.5056**
	Seasonal	**0.9965**	0.9949	59.6527	92.8238
**SGLT2i**	Base	0.9971	0.9968	**144.8584**	**156.5659**
	Seasonal	**0.9979**	**0.9970**	150.0100	183.1811

ACEI: angiotensin-converting enzyme inhibitor. ARB: angiotensin receptor blocker. ARNI: angiotensin receptor-neprilysin inhibitor. MRA: mineralocorticoid receptor antagonist. SGLT2i: sodium glucose cotransporter 2 inhibitor.
6 parameters for base model; 17 parameters for seasonal model. Bold values indicate better fit. Adjusted R^2 varies by medication; AIC and BIC predominantly favor the parsimony in the base model.

Table S1B: Model Diagnostics of Seasonal vs Non-seasonal Models for Visit Data

Outcome	R^2 Base Model	R^2 With Seasonal Controls	Improvement
**Primary Care**			
All Primary Care Visits	0.869	**0.911**	+0.042
Face-to-Face	0.617	**0.835**	+0.218
Telehealth	0.906	**0.923**	+0.017
CVT	0.908	**0.935**	+0.027
Video	0.774	**0.820**	+0.046
Phone	0.908	**0.925**	+0.017
**Cardiology**			
All Cardiology Visits	0.683	**0.813**	+0.130
Face-to-Face	0.819	**0.910**	+0.091
Telehealth	0.902	**0.913**	+0.011
CVT	0.906	**0.938**	+0.032
Video	0.788	**0.826**	+0.038
Phone	0.904	**0.915**	+0.011

6 parameters for base model; 17 parameters for seasonal model. Bold values indicate better fit. R^2 universally support seasonal model.

Table S1C: Model Diagnostics of Seasonal vs Non-seasonal Models for Clinical Outcomes

Outcome	Model	R^2	R^2 adj	aic	bic
**All-Cause Hospitalization**	Base	0.820	0.806	-712.67	**-699.01**
	Seasonal	**0.896**	**0.866**	**-730.20**	-691.49
**Heart Failure Hospitalization**	Base	0.633	0.605	-906.62	-892.96
	Seasonal	**0.812**	**0.757**	**-932.89**	**-894.19**
**Mortality**	Base	0.565	0.532	-729.12	-715.46
	Seasonal	**0.874**	**0.837**	**-796.10**	**-757.40**
**COVID Hospitalization**	Base	0.155	0.108	-697.68	**-689.44**
	Seasonal	**0.608**	**0.480**	**-720.24**	-689.34

Bold values indicate better fit. 6 parameters for base model; 17 parameters for seasonal model, except for COVID hospitalizations, which eliminated the pre-COVID period (15 parameters). R^2 universally support seasonal model. AIC and BIC predominantly favor seasonal model.

medRxiv preprint doi: https://doi.org/10.1101/2025.10.13.25337954; this version posted October 15, 2025. The copyright holder for this preprint
(which was not certified by peer review) is the author/funder, who has granted medRxiv a license to display the preprint in perpetuity.
It is made available under a CC-BY 4.0 International license .

Table S2: Full Regression Results for Interrupted Time Series for Medication Utilization

	ACE ARB ARNI	ARNI	Beta-blocker	MRA	SGLT2i
**Pre-COVID Trend (per period)**					
Coeff.	0.0085	0.40***	0.041***	0.14***	0.29***
95% CI	[-0.019, 0.036]	[0.38, 0.42]	[0.020, 0.062]	[0.13, 0.15]	[0.26, 0.33]
**Early COVID (Period 2)**					
Level Change	-1.76*	-3.08**	0.88**	-2.03**	-6.17***
95% CI	[-3.47, -0.062]	[-5.13, -1.04]	[0.34, 1.42]	[-3.33, -0.73]	[-9.00, -3.34]
Trend Change	0.12*	0.22**	-0.078***	0.14**	0.48***
95% CI	[0.021, 0.22]	[0.089, 0.34]	[-0.12, -0.037]	[0.062, 0.22]	[0.30, 0.65]
**Late COVID (Period 3)**					
Level Change	-1.77***	-4.29**	-3.93***	-7.18***	-15.18***
95% CI	[-2.25, -1.28]	[-6.72, -1.85]	[-5.97, -1.90]	[-8.49, -5.87]	[-19.15, -11.21]
Trend Change	0.14***	0.36***	0.093**	0.33***	0.97***
95% CI	[0.12, 0.17]	[0.30, 0.43]	[0.040, 0.15]	[0.29, 0.36]	[0.86, 1.08]

ACEI: angiotensin-converting enzyme inhibitor. ARB: angiotensin receptor blocker. ARNI: angiotensin receptor-neprilysin inhibitor. MRA: mineralocorticoid receptor antagonist. SGLT2i: sodium glucose cotransporter 2 inhibitor.

Presented results are from regression models with Newey-West robust standard errors to account for heteroskedasticity and autocorrelation as found in the time series data. Reference period: Pre-COVID (period 1).

*p < 0.05, **p < 0.01, ***p < 0.001 (based on confidence intervals not including zero). Level changes represent immediate shifts at period transitions; trend changes represent changes in the slope (rate of change per period).

medRxiv preprint doi: https://doi.org/10.1101/2025.10.13.25337954; this version posted October 15, 2025. The copyright holder for this preprint (which was not certified by peer review) is the author/funder, who has granted medRxiv a license to display the preprint in perpetuity.
It is made available under a CC-BY 4.0 International license .

Table S3: Full Regression Results for Interrupted Time Series for Primary Care Visits

Variable	All	Telehealth	Face-to-face	CVT	Video	Phone
**Time Trends**						
**Base Trend (monthly)**	-0.000<br>*(0.000)*	-0.000<br>*(0.000)*	-0.000<br>*(0.000)*	0.000**<br>*(0.000)*	0.000<br>*(0.000)*	-0.000<br>*(0.000)*
	Period Effects (Level Changes) – ref. Pre COVID					
**Early COVID**	0.116***<br>*(0.031)*	0.182***<br>(0.032)	-0.066***<br>(0.010)	-0.000*<br>*(0.000)*	-0.007***<br>(0.001)	0.189***<br>(0.032)
**Late COVID**	0.062***<br>(0.006)	0.086***<br>(0.007)	-0.024***<br>(0.004)	-0.001***<br>*(0.000)*	0.005***<br>(0.001)	0.082***<br>(0.007)
	Trend Changes (Period × Time Interactions) – ref. Pre-COVID x Time					
**Early COVID × Time**	-0.002<br>*(0.001)*	-0.004**<br>*(0.001)*	0.002***<br>*(0.000)*	-0.000<br>*(0.000)*	0.000***<br>*(0.000)*	-0.005***<br>*(0.001)*
**Late COVID × Time**	-0.001**<br>*(0.000)*	-0.001***<br>*(0.000)*	0.000<br>*(0.000)*	0.000<br>*(0.000)*	-0.000<br>*(0.000)*	-0.001***<br>*(0.000)*
	Seasonal Controls – ref. January					
**February**	-0.017***<br>*(0.003)*	-0.011***<br>*(0.002)*	-0.005***<br>*(0.001)*	-0.000***<br>*(0.000)*	-0.000<br>*(0.000)*	-0.011***<br>*(0.002)*
**March**	0.004<br>*(0.011)*	-0.002<br>*(0.013)*	0.006*<br>*(0.003)*	0.000<br>*(0.000)*	0.000<br>*(0.000)*	-0.002<br>*(0.013)*
**April**	0.005<br>*(0.003)*	0.006<br>*(0.006)*	-0.001<br>*(0.004)*	-0.000<br>*(0.000)*	-0.000<br>*(0.001)*	0.007<br>*(0.006)*
**May**	0.001<br>*(0.004)*	0.002<br>*(0.004)*	-0.001<br>*(0.003)*	-0.000<br>*(0.000)*	-0.001<br>*(0.001)*	0.003<br>*(0.004)*
**June**	-0.002<br>*(0.005)*	-0.001<br>*(0.006)*	-0.001<br>*(0.001)*	-0.000<br>*(0.000)*	-0.001<br>*(0.001)*	0.000<br>*(0.006)*
**July**	-0.002<br>*(0.006)*	-0.002<br>*(0.005)*	-0.001<br>*(0.002)*	-0.000<br>*(0.000)*	-0.001<br>*(0.001)*	-0.000<br>*(0.005)*
**August**	0.009**<br>*(0.003)*	0.005<br>*(0.004)*	0.004**<br>*(0.002)*	0.000<br>*(0.000)*	-0.001<br>*(0.001)*	0.006<br>*(0.004)*
**September**	-0.000<br>*(0.005)*	-0.006<br>*(0.004)*	0.006*<br>*(0.003)*	-0.000<br>*(0.000)*	-0.001<br>*(0.001)*	-0.006<br>*(0.003)*
**October**	0.013*<br>*(0.005)*	-0.003<br>*(0.004)*	0.016***<br>*(0.003)*	-0.000<br>*(0.000)*	-0.001<br>*(0.001)*	-0.002<br>*(0.004)*
**November**	-0.011*<br>*(0.005)*	-0.014*<br>*(0.006)*	0.002<br>*(0.002)*	-0.000**<br>*(0.000)*	-0.001<br>*(0.001)*	-0.013*<br>*(0.005)*
**December**	-0.005<br>*(0.004)*	-0.005<br>*(0.003)*	-0.000<br>*(0.002)*	-0.000**<br>*(0.000)*	-0.001<br>*(0.001)*	-0.004<br>*(0.004)*

Presented results are from regression models with Newey-West robust standard errors to account for heteroskedasticity and autocorrelation as found in the time series data. Reference period: Pre-COVID (period 1).

*p < 0.05, **p < 0.01, ***p < 0.001 (based on confidence intervals). Standard errors in parentheses. Level changes represent immediate shifts at period transitions; trend changes represent changes in the slope (rate of change per period).

medRxiv preprint doi: https://doi.org/10.1101/2025.10.13.25337954; this version posted October 15, 2025. The copyright holder for this preprint (which was not certified by peer review) is the author/funder, who has granted medRxiv a license to display the preprint in perpetuity.
It is made available under a CC-BY 4.0 International license .

Table S4: Full Regression Results of Interrupted Time Series for Cardiology Visits

Variable	All	Telehealth	Face-to-face	CVT	Video	Phone
**Time Trends**						
**Base Trend (monthly)**	-0.000*** *(0.000)*	0.000 *(0.000)*	-0.000** *(0.000)*	0.000 *(0.000)*	0.000 *(0.000)*	-0.000 *(0.000)*
	Period Effects (Level Changes) – ref. Pre COVID					
**Early COVID**	0.032** (0.011)	0.086*** (0.013)	-0.054*** (0.006)	-0.001*** *(0.000)*	-0.003*** (0.000)	0.090*** (0.013)
**Late COVID**	-0.007* (0.003)	0.013*** (0.002)	-0.020*** (0.002)	-0.001*** *(0.000)*	0.002*** (0.000)	0.012*** (0.002)
	Trend Changes (Period × Time Interactions) – ref. Pre-COVID x Time					
**Early COVID × Time**	-0.000 *(0.000)*	-0.002*** *(0.001)*	0.002*** *(0.000)*	0.000** *(0.000)*	0.000*** *(0.000)*	-0.002*** *(0.001)*
**Late COVID × Time**	0.001*** *(0.000)*	-0.000 *(0.000)*	0.001*** *(0.000)*	0.000*** *(0.000)*	-0.000 *(0.000)*	-0.000 *(0.000)*
	Seasonal Controls (ref. January)_					
**February**	-0.004*** *(0.001)*	-0.001*** *(0.000)*	-0.003** *(0.001)*	-0.000 *(0.000)*	-0.000 *(0.000)*	-0.001*** *(0.000)*
**March**	0.003 *(0.004)*	-0.004 *(0.005)*	0.007*** *(0.002)*	0.000 *(0.000)*	-0.000 *(0.000)*	-0.004 *(0.005)*
**April**	0.004** *(0.001)*	0.002 *(0.003)*	0.002 *(0.002)*	0.000 *(0.000)*	-0.000 *(0.000)*	0.002 *(0.003)*
**May**	0.003 *(0.002)*	0.001 *(0.002)*	0.002 *(0.002)*	0.000* *(0.000)*	-0.000 *(0.000)*	0.001 *(0.002)*
**June**	0.001 *(0.002)*	-0.000 *(0.001)*	0.001 *(0.001)*	0.000* *(0.000)*	-0.000 *(0.000)*	-0.000 *(0.001)*
**July**	-0.001 *(0.002)*	-0.001 *(0.001)*	0.000 *(0.001)*	-0.000 *(0.000)*	-0.000 *(0.000)*	-0.001 *(0.001)*
**August**	0.004** *(0.001)*	0.000 *(0.001)*	0.004*** *(0.001)*	0.000** *(0.000)*	-0.000 *(0.000)*	0.000 *(0.001)*
**September**	0.000 *(0.002)*	-0.002 *(0.001)*	0.002 *(0.001)*	0.000 *(0.000)*	-0.000 *(0.000)*	-0.001 *(0.001)*
**October**	0.002 *(0.001)*	-0.001 *(0.001)*	0.003* *(0.001)*	0.000** *(0.000)*	-0.000 *(0.000)*	-0.001 *(0.001)*
**November**	-0.005** *(0.002)*	-0.003* *(0.002)*	-0.002 *(0.001)*	0.000 *(0.000)*	-0.000 *(0.000)*	-0.003* *(0.001)*
**December**	-0.004* *(0.002)*	-0.002*** *(0.001)*	-0.002 *(0.002)*	-0.000 *(0.000)*	-0.000 *(0.000)*	-0.002** *(0.001)*

Presented results are from regression models with Newey-West robust standard errors to account for heteroskedasticity and autocorrelation as found in the time series data. Reference period: Pre-COVID (period 1).

*p < 0.05, **p < 0.01, ***p < 0.001 (based on confidence intervals). Standard errors in parentheses. Level changes represent immediate shifts at period transitions; trend changes represent changes in the slope (rate of change per period).

medRxiv preprint doi: https://doi.org/10.1101/2025.10.13.25337954; this version posted October 15, 2025. The copyright holder for this preprint (which was not certified by peer review) is the author/funder, who has granted medRxiv a license to display the preprint in perpetuity.
It is made available under a CC-BY 4.0 International license .

Table S5: Full Regression Results of Interrupted Time Series for Hospitalization and Mortality

Variable	All-cause Hospitalizations	Heart Failure Hospitalizations	Mortality	COVID-19 Hospitalizations
Base Trend (monthly)	-0.0001	-0.0000	0.0001	-0.0000
	(0.0001)	(0.0000)	(0.0001)	(0.0000)
Period Effects (Level Changes) – ref. Pre COVID				
Early COVID	-0.0190***	-0.0031***	0.0024*	0.0000
	(0.0026)	(0.0005)	(0.0011)	(.)
Late COVID	-0.0094***	-0.0015***	0.0056***	0.0007
	(0.0008)	(0.0002)	(0.0011)	(0.0004)
**Trend Changes (Period × Time Interactions) – ref. Pre-COVID x Time**				
Early COVID × Time	0.0005***	0.0001***	-0.0000	0.0000
	(0.0001)	(0.0000)	(0.0001)	(.)
Late COVID × Time	0.0001	0.0000	-0.0001	-0.0000
	(0.0001)	(0.0000)	(0.0001)	(0.0000)
Monthly Effects				
**February**	-0.0026***	-0.0004*	-0.0026***	-0.0012*
	(0.0006)	(0.0002)	(0.0005)	(0.0006)
**March**	0.0015	0.0003	-0.0023**	-0.0016*
	(0.0008)	(0.0002)	(0.0008)	(0.0006)
**April**	-0.0008	-0.0003	-0.0032***	-0.0015*
	(0.0013)	(0.0003)	(0.0008)	(0.0006)
**May**	0.0003	-0.0002	-0.0033***	-0.0015*
	(0.0007)	(0.0002)	(0.0008)	(0.0006)
**June**	-0.0005	-0.0005*	-0.0043***	-0.0015*
	(0.0007)	(0.0002)	(0.0007)	(0.0006)
**July**	-0.0007	-0.0007***	-0.0033***	-0.0012*
	(0.0008)	(0.0002)	(0.0007)	(0.0006)
**August**	0.0001	-0.0005*	-0.0036***	-0.0010
	(0.0006)	(0.0002)	(0.0007)	(0.0006)
**September**	-0.0014	-0.0008***	-0.0038***	-0.0011
	(0.0008)	(0.0002)	(0.0007)	(0.0006)
**October**	-0.0001	-0.0002	-0.0029***	-0.0012*
	(0.0008)	(0.0003)	(0.0006)	(0.0006)
**November**	-0.0020**	-0.0005*	-0.0027***	-0.0010
	(0.0006)	(0.0002)	(0.0006)	(0.0006)
**December**	-0.0004	-0.0002	-0.0007	-0.0006
	(0.0006)	(0.0002)	(0.0006)	(0.0007)

Presented results are from regression models with Newey-West robust standard errors to account for heteroskedasticity and autocorrelation as found in the time series data. Reference period: Pre-COVID (period 1).

*p < 0.05, **p < 0.01, ***p < 0.001 (based on confidence intervals not including zero). Level changes represent immediate shifts at period transitions; trend changes represent changes in the slope (rate of change per period).

medRxiv preprint doi: https://doi.org/10.1101/2025.10.13.25337954; this version posted October 15, 2025. The copyright holder for this preprint (which was not certified by peer review) is the author/funder, who has granted medRxiv a license to display the preprint in perpetuity.
It is made available under a CC-BY 4.0 International license .

Table S6: Demographics and Comorbidities for Imputed Datasets and Complete Cases

Characteristic	Multiple Imputation (Primary)	Complete Case (Sensitivity)
**Sample Size**	N = 210535	N = 139169
**DEMOGRAPHICS**		
**Age, years**	73.1 ± 10.6	72.5 ± 10.2
**Female sex**	4639 (2.2%)	3135 (2.3%)
**Race/Ethnicity**		
**White**	146221 (69.5%)	97082 (69.8%)
**Black**	40172 (19.1%)	27724 (19.9%)
**Hispanic**	9143 (4.3%)	4899 (3.5%)
**Other**	14999 (7.1%)	9464 (6.8%)
**VITAL SIGNS AND LABS**		
**BMI, kg/m²**	29.8 ± 6.7	30.0 ± 6.7
**Heart rate, bpm**	75.0 ± 14.4	75.1 ± 14.5
**Systolic BP, mmHg**	126.5 ± 19.4	126.6 ± 19.5
**Diastolic BP, mmHg**	72.3 ± 11.5	72.4 ± 11.5
**Hemoglobin, g/dL**	13.0 ± 2.2	13.0 ± 2.2
**eGFR, mL/min/1.73m²**	63.0 ± 27.9	62.3 ± 27.8
**Ejection Fraction, %**	32.5 ± 14.5	32.7 ± 14.6
**COMORBIDITIES**		
**Atrial fibrillation**	91798 (43.6%)	63482 (45.6%)
**Coronary artery disease**	151031 (71.7%)	103649 (74.5%)
**Chronic kidney disease**	111959 (53.2%)	78141 (56.1%)
**COPD**	18067 (8.6%)	12892 (9.3%)
**Diabetes mellitus**	129413 (61.5%)	88702 (63.7%)
**Hyperlipidemia**	161487 (76.7%)	112401 (80.8%)
**Hypertension**	187912 (89.3%)	127624 (91.7%)
**Cerebrovascular accident**	31063 (14.8%)	21831 (15.7%)
**Alcohol use disorder**	25552 (12.1%)	18158 (13.0%)
**Substance use disorder**	18328 (8.7%)	13198 (9.5%)
**Smoking**	39741 (18.9%)	28020 (20.1%)
**Depression**	54828 (26.0%)	38411 (27.6%)
**Peripheral artery disease**	107021 (50.8%)	74716 (53.7%)
**Cancer**	37158 (17.6%)	25821 (18.6%)
**OUTCOMES**		
**Death**	92459 (43.9%)	68172 (49.0%)
**First hospitalization**	61380 (29.2%)	49630 (35.7%)
**Composite endpoint**	119562 (56.8%)	89602 (64.4%)

BMI, body mass index; BP, blood pressure; COPD, chronic obstructive pulmonary disease; eGFR, estimated glomerular filtration rate.

Baseline characteristics under the Multiple Imputation header are presented for the first imputed dataset (m=1), representative of all imputed datasets. Complete cases included patients with valid survival times, event status, and no missing values in covariates after survival data preparation (n=139,169; 66.1%). Baseline characteristics imputed closest to cohort entry, which may not reflect patients' clinical status throughout the 6 years of follow up, particularly given the COVID-related disruptions to the healthcare system.

medRxiv preprint doi: https://doi.org/10.1101/2025.10.13.25337954; this version posted October 15, 2025. The copyright holder for this preprint (which was not certified by peer review) is the author/funder, who has granted medRxiv a license to display the preprint in perpetuity.
It is made available under a CC-BY 4.0 International license .

Table S7: Sensitivity Analysis**:** Cox Proportional Hazard Models for Mortality and Hospitalization

Variable	All-Cause Mortality HR (95% CI)	All-Cause Hospitalization HR (95% CI)	Composite Endpoint HR (95% CI)
**Demographics**			
**Age at entry (per year)**	1.034 (1.033-1.035)***	0.997 (0.996-0.999)***	1.015 (1.014-1.016)***
**Female sex**	0.804 (0.757-0.854)***	0.907 (0.853-0.965)**	0.885 (0.843-0.929)***
**Race/Ethnicity**			
**White (reference)**	1	1	1
**Black**	0.964 (0.944-0.984)**	1.371 (1.340-1.402)***	1.148 (1.127-1.168)***
**Hispanic**	0.900 (0.862-0.939)***	1.168 (1.115-1.224)***	1.021 (0.984-1.058)
**Other**	0.933 (0.904-0.962)***	0.940 (0.906-0.976)**	0.955 (0.929-0.981)**
**Geographic Region**			
**Midwest (reference)**	1	1	1
**Northeast**	0.981 (0.955-1.006)	0.978 (0.948-1.009)	0.984 (0.962-1.007)
**South**	1.017 (0.998-1.037)	0.971 (0.950-0.993)*	0.989 (0.972-1.005)
**West**	1.042 (1.018-1.066)**	0.978 (0.951-1.006)	1.007 (0.987-1.028)
**Clinical Parameters**			
**BMI (per kg/m²)**	0.991 (0.990-0.992)***	1.000 (0.998-1.001)	0.998 (0.996-0.999)***
**Pulse (per bpm)**	1.001 (1.000-1.001)*	0.998 (0.998-0.999)***	0.999 (0.999-1.000)**
**Systolic BP (per mmHg)**	1.003 (1.003-1.004)***	1.003 (1.003-1.004)***	1.004 (1.003-1.004)***
**Diastolic BP (per mmHg)**	0.995 (0.994-0.996)***	1.000 (0.999-1.001)	0.998 (0.997-0.998)***
**Hemoglobin (per g/dL)**	0.978 (0.975-0.982)***	1.009 (1.005-1.013)***	1.000 (0.997-1.003)
**eGFR (per mL/min/1.73m²)**	0.997 (0.996-0.997)***	1.000 (1.000-1.000)*	0.999 (0.998-0.999)***
**LVEF**	0.997 (0.997-0.998)***	0.984 (0.984-0.985)***	0.992 (0.992-0.993)***
**Comorbidities**			
**Atrial fibrillation**	1.082 (1.065-1.099)***	1.095 (1.075-1.115)***	1.078 (1.064-1.093)***
**Coronary artery disease**	1.057 (1.037-1.077)***	1.208 (1.182-1.235)***	1.098 (1.081-1.116)***
**Chronic kidney disease**	1.130 (1.106-1.155)***	1.150 (1.120-1.180)***	1.126 (1.105-1.148)***
**COPD**	1.153 (1.125-1.182)***	1.067 (1.035-1.100)***	1.097 (1.072-1.121)***
**Diabetes mellitus**	1.050 (1.028-1.072)***	1.009 (0.985-1.034)	1.031 (1.013-1.050)**
**Hyperlipidemia**	0.924 (0.906-0.943)***	1.024 (0.999-1.049)	0.957 (0.940-0.974)***
**Hypertension**	0.994 (0.964-1.025)	1.180 (1.137-1.225)***	1.070 (1.042-1.098)***
**Cerebrovascular disease**	1.098 (1.077-1.121)***	1.090 (1.065-1.117)***	1.088 (1.069-1.107)***
**Alcohol use disorder**	1.115 (1.087-1.144)***	1.128 (1.097-1.160)***	1.116 (1.092-1.140)***
**Substance use disorder**	1.135 (1.101-1.169)***	1.230 (1.193-1.269)***	1.198 (1.168-1.228)***
**Smoking**	1.213 (1.188-1.238)***	1.151 (1.125-1.178)***	1.164 (1.144-1.185)***
**Depression**	1.056 (1.037-1.075)***	1.140 (1.118-1.163)***	1.103 (1.087-1.120)***
**Peripheral artery disease**	1.168 (1.146-1.190)***	1.153 (1.127-1.179)***	1.135 (1.117-1.154)***
**Cancer**	1.052 (1.032-1.072)***	1.106 (1.081-1.132)***	1.043 (1.026-1.061)***
**Model Statistics**			
**C-statistic**	0.643	0.618	0.595

BMI, body mass index; BP, blood pressure; COPD, chronic obstructive pulmonary disease; eGFR, estimated glomerular filtration rate; HR, hazard ratio; LVEF, left ventricular ejection fraction.

Statistical significance: *P < 0.05, **P < 0.01, ***P < 0.001

Note: HR results from complete case analysis (N = 139,169); C statistic from same dataset. Models adjusted for all variables shown. Composite endpoint defined as time to first occurrence of all-cause mortality or heart failure hospitalization. White race serves as the reference category for race/ethnicity comparisons; Midwest serves as the reference category for geographic comparisons.

medRxiv preprint doi: https://doi.org/10.1101/2025.10.13.25337954; this version posted October 15, 2025. The copyright holder for this preprint (which was not certified by peer review) is the author/funder, who has granted medRxiv a license to display the preprint in perpetuity.
It is made available under a CC-BY 4.0 International license .

Table S8: Estimated Number of Patients for GDMT Measures

Table S8A: COVID Onset Impact (Early COVID compared to Pre-COVID)

Medication	HFrEF + HFimpEF on med (patients)	95% CI	HFrEF (patients)	95% CI
**ACE ARB ARNI**	-587	(-1,389 to 216)	-497	(-1,178 to 183)
**ARNI**	-832	(-1,575 to -89)	-825	(-1,561 to -89)
**Beta-blocker**	190	(-189 to 570)	160	(-159 to 479)
**MRA**	-596	(-1,108 to -84)	-570	(-1,059 to -81)
**SGLT2i**	-1293	(-2,349 to -236)	-1273	(-2,313 to -232)

Table S8B: Late COVID Impact (Late COVID compared to Pre-COVID)

Medication	HFrEF + HFimpEF on med (patients)	95% CI	HFrEF (patients)	95% CI
**ACE ARB ARNI**	493	(-99 to 1,085)	419	(-84 to 923)
**ARNI**	1730	(517 to 2,943)	1698	(507 to 2,888)
**Beta-blocker**	-1613	(-3,259 to 34)	-1372	(-2,772 to 29)
**MRA**	-714	(-1,232 to -197)	-679	(-1,171 to -187)
**SGLT2i**	1821	(49 to 3,593)	1764	(47 to 3,481)

Table S8C: Counterfactual Trends (Expected vs Observed by Study End Date)

Medication	HFrEF + HFimpEF on med (patients)	95% CI	HFrEF (patients)	95% CI
**ACE ARB ARNI**	+462	(-960 to 1,884)	+384	(-799 to 1,566)
**ARNI**	+19,408	(18,401 to 20,415)	+17,935	(17,004 to 18,867)
**Beta-blocker**	+3,648	(1,836 to 5,459)	+3,033	(1,525 to 4,541)
**MRA**	+6,787	(6,083 to 7,490)	+6,214	(5,569 to 6,859)
**SGLT2i**	+14,854	(13,056 to 16,651)	+13,018	(11,441 to 14,595)

ACEI: angiotensin-converting enzyme inhibitor. ARB: angiotensin receptor blocker. ARNI: angiotensin receptor-neprilysin inhibitor. HFimpEF: heart failure with improved ejection fraction. HFrEF: heart failure with reduced ejection fraction. MRA: mineralocorticoid receptor antagonist. SGLT2i: sodium glucose cotransporter 2 inhibitor.

*$p < 0.05$, (based on confidence intervals). Patient counts represent estimated impact on study population. Counterfactual analysis compares observed end-of-study rates to projected pre-COVID trends (Pre-COVID trend coefficient x 52 months). Coefficients found in Table 2.2.

medRxiv preprint doi: https://doi.org/10.1101/2025.10.13.25337954; this version posted October 15, 2025. The copyright holder for this preprint
(which was not certified by peer review) is the author/funder, who has granted medRxiv a license to display the preprint in perpetuity.
It is made available under a CC-BY 4.0 International license .

Table S9: ICD Codes

Atrial fibrillation

I48.0	I48.11	I48.19	I48.20	I48.21	I48.3	I48.4
I48.91	I48.92	427.31	427.32			

Ischemic Heart Disease

I21.X	I22.X	I20.X	I23.0	I23.1	I23.2	I23.3
I23.6	I23.7	I23.8	I24.X	I25.X	Z95.1	Z95.5
Z98.61	410.X	411.X	412	413.X	414.X	

Chronic Kidney Disease

N18.4	N18.5	N18.6	T81.502X	T81.512X	T81.522X
T81.532X	T81.592X	T82.41X	T85.611X	T85.621X	T85.631X
T85.691X	T85.571X	Y62.2	Z49.X	Z91.15	Z9.92
N18.3	N19	I12.X	I13.X	Z91.15	E08.2X
E09.2X	E10.2X	E11.2X	E13.2X	585.3	585.4
585.5	585.6	585.9	586		

Chronic Obstructive Pulmonary Disease

J41.X	J42	J43.X	J44.0	491.X	491.2X	491.8
491.9	492.8	496				

Diabetes Mellitus

E08.X	E09.X	E10.X	E11.X	E13.X	O24.X	250.X

Hyperlipidemia

E78

Hypertension

I10	I11.X	I12.X	I13.X	I15.X	I16.X	401.X
402.X	403.X	404.X	405.X			

Cerebrovascular Disease

I63.X	I97.81X	I97.82X	433.01	433.11	433.2
433.31	433.81	433.91	434.401	434.11	434.91

Alcohol Related Disorders

F10.1X	F10.12X	F10.13X	F10.14	F10.15X	F10.18X	
F10.19	F10.2X	F10.22X	F10.23X	F10.24	F10.25X	
F10.26	F10.27	F10.28X	F10.29	K70.X	T51.0X1A	
T510X1D	T510X1S	T51.0X2A	T51.0X2D	T51.0X2S	T51.0X3A	
T51.0X3D	T51.0X3S	T51.0X4A	T51.0X4D	T51.0X4S	Z7141	291.X
303.0	305.0X	357.5	425.5	535.3X	571.0	571.1
571.2	571.3	980.8	980.8	V11.3		

medRxiv preprint doi: https://doi.org/10.1101/2025.10.13.25337954; this version posted October 15, 2025. The copyright holder for this preprint (which was not certified by peer review) is the author/funder, who has granted medRxiv a license to display the preprint in perpetuity.
It is made available under a CC-BY 4.0 International license .

Substance Abuse

F11.1X	F11.2X	F12.1X	F12.2X	F13.1X	F13.2X	
F14.1X	F14.2X	F15.1X	F15.2X	F16.1X	F16.2X	
F18.1X	F18.2X	F19.1X	F19.2X	291.X	292.X	303.X
304.X	305.2X	305.3X	305.4X	305.5X	305.6X	
305.7X	305.8X	305.9X				

Tobacco use disorder

F17

Depression

F31.1X	F31.4	F31.5	F32.X	F32.8XF	32.9
F33.4X	F33.8	F33.9	F41.2	F43.2	296.2X
296.30	296.31	296.32	296.33	296.34	296.36
296.5X	300.4	309.0	309.1	309.28	311

Peripheral Arterial Disease

E08.51	E08.52	E08.59	E09.5X	E10.5X	E11.5X	
E13.5X	I70.X	I71.X	I73.1	I73.8X	I73.9	I77.1
I79.X	K55.1	K55.8	K55.9	Z95.82X	930	931
437.3	443.1	447.1	557.1	557.9	V43.4	440.X
441.X	443.2X	443.8X	443.9	447.1	249.7	
250.7X	I74.X	444.0X	444.2X	444.8X	444.9	I75.X
445.X	I77.7X	443.2X	I77.2	447.2		

Cancer

C01	C02.X	C03.X	C04.X	C05.X	C06.X	C07
C08.X	C09.X	C10.X	C11.X	C12	C13.X	C14.X
C15.X	C16.X	C17.X	C18.X	C19	C20	C21.X
C22.X	C23.X	C24.X	C25.X	C26.X	C30.X	C31.X
C32.X	C33	C34.X	C37	C38.X	C39.X	C40.X
C41.X	C43.X	C45.X	C46.X	C47.X	C48.X	C49.X
C50.X	C4A.X	C51.X	C52	C53.X	C54.X	C55
C56.X	C57.X	C58	C60.X	C61	C62.X	C63.X
C64.X	C65.X	C66.X	C67.X	C68.X	C69.X	C71.X
C72.X	C73	C74.X	C75.X	C76.X	C77.X	C78.X
C79.X	C7A.X	C7B.X	C80.X	C81.X	C82.X	C83.X
C84.X	C85.X	C86.X	C88.X	C90.X	C91.X	C92.X
C93.X	C94.X	C95.X	C96.X	D03.X	E34.0	141.X
142.X	143.X	144.X	145.X	146.X	147.X	148.X
149.X	150.X	151.X	152.X	153.X	154.X	155.X
156.X	157.X	158.X	159.X	160.X	161.X	162.X
163.X	164.X	165.X	170.X	171.X	172.X	174.X
175.X	176.X	179	180.X	181	182.X	183.X
184.X	185	186.X	187.X	188.X	189.X	190.X
191.X	192.X	193	194.X	195.X	196.X	197.X

medRxiv preprint doi: https://doi.org/10.1101/2025.10.13.25337954; this version posted October 15, 2025. The copyright holder for this preprint (which was not certified by peer review) is the author/funder, who has granted medRxiv a license to display the preprint in perpetuity.
It is made available under a CC-BY 4.0 International license .

198.X	199.X	200.X	201.X	202.X	203.X	204.X
205.X	206.X	207.X	208.X	209.X	225.X	227.X
228.02	237.0	237.1	237.3	237.5	237.6	
237.7X	237.9	239.6	259.2	759.5	759.6	

medRxiv preprint doi: https://doi.org/10.1101/2025.10.13.25337954; this version posted October 15, 2025. The copyright holder for this preprint (which was not certified by peer review) is the author/funder, who has granted medRxiv a license to display the preprint in perpetuity.
It is made available under a CC-BY 4.0 International license .

Figure S1: Selection of final cohort for analyses

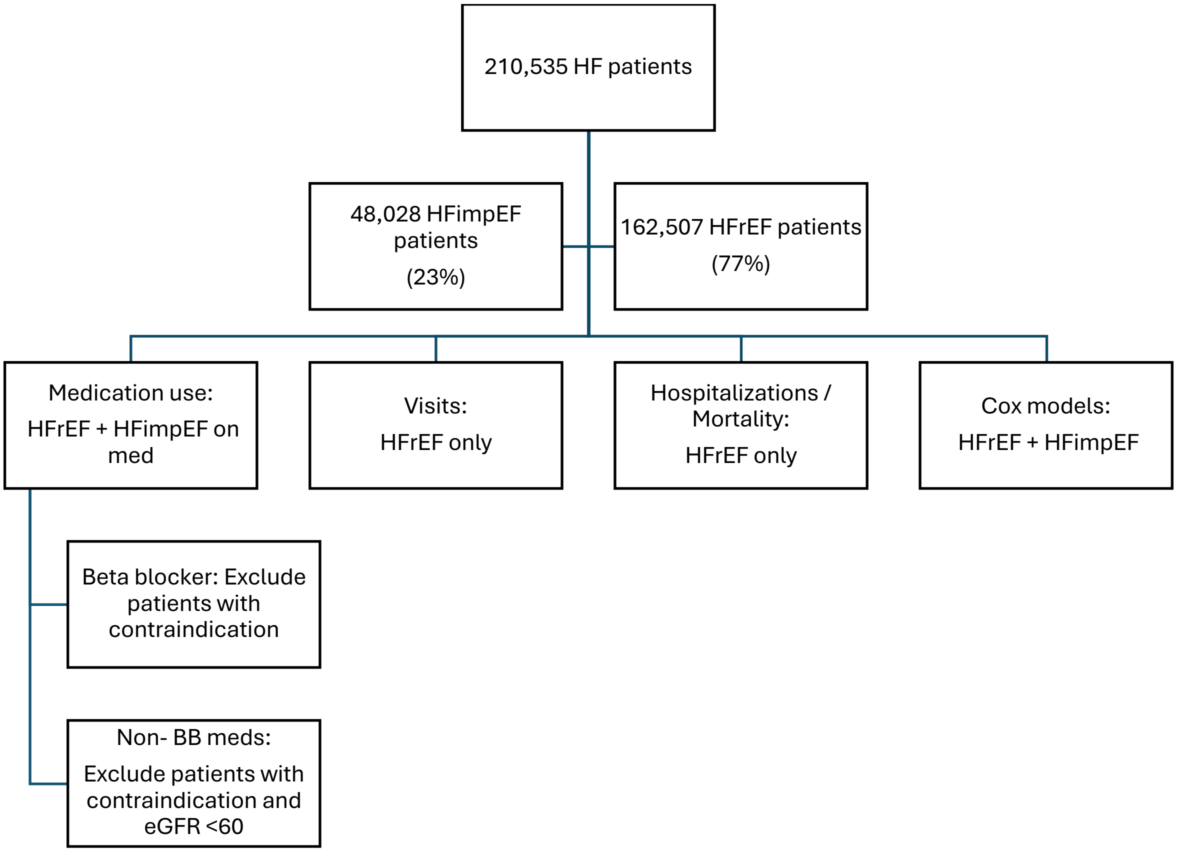

BB: Beta blocker. eGFR: estimated glomerular filtration rate. HF: heart failure. HFimpEF: heart failure with improved ejection fraction. HFrEF: heart failure with reduced ejection fraction.

medRxiv preprint doi: https://doi.org/10.1101/2025.10.13.25337954; this version posted October 15, 2025. The copyright holder for this preprint (which was not certified by peer review) is the author/funder, who has granted medRxiv a license to display the preprint in perpetuity.
It is made available under a CC-BY 4.0 International license .

Figure S2: Optimal Breakpoint Selection Based on Variance Weighted Averages of Combined F-statistic for Hospitalizations and Mortality

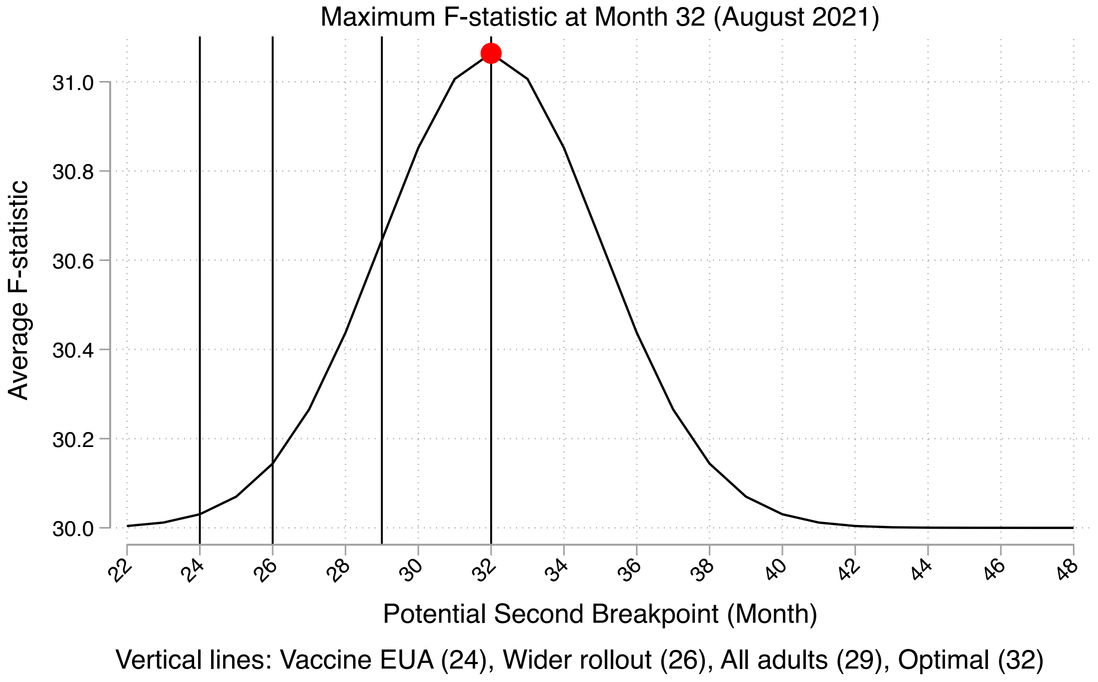

March 2020 marked the declaration of a National Pandemic Emergency in the United States so was chosen as breakpoint 1.[20]
Optimal breakpoint 2 was determined using structural break detection methods, testing all potential breakpoints from month 22 to 48 and noting August 2021 as the superior model fit across outcomes;[21] specifically, systematic F-statistic optimization using variance weighted averages noted the highest combined F-statistic at month 32. Clinically this corresponded to the peak of the Delta variant, with the highest COVID hospitalizations since the initial surge as well as increased vaccine availability and eligibility for the general population.[22]

medRxiv preprint doi: https://doi.org/10.1101/2025.10.13.25337954; this version posted October 15, 2025. The copyright holder for this preprint (which was not certified by peer review) is the author/funder, who has granted medRxiv a license to display the preprint in perpetuity.
It is made available under a CC-BY 4.0 International license .

Figure S3: Individual Heart Failure Medication Utilization Trends Between 2019 and 2024

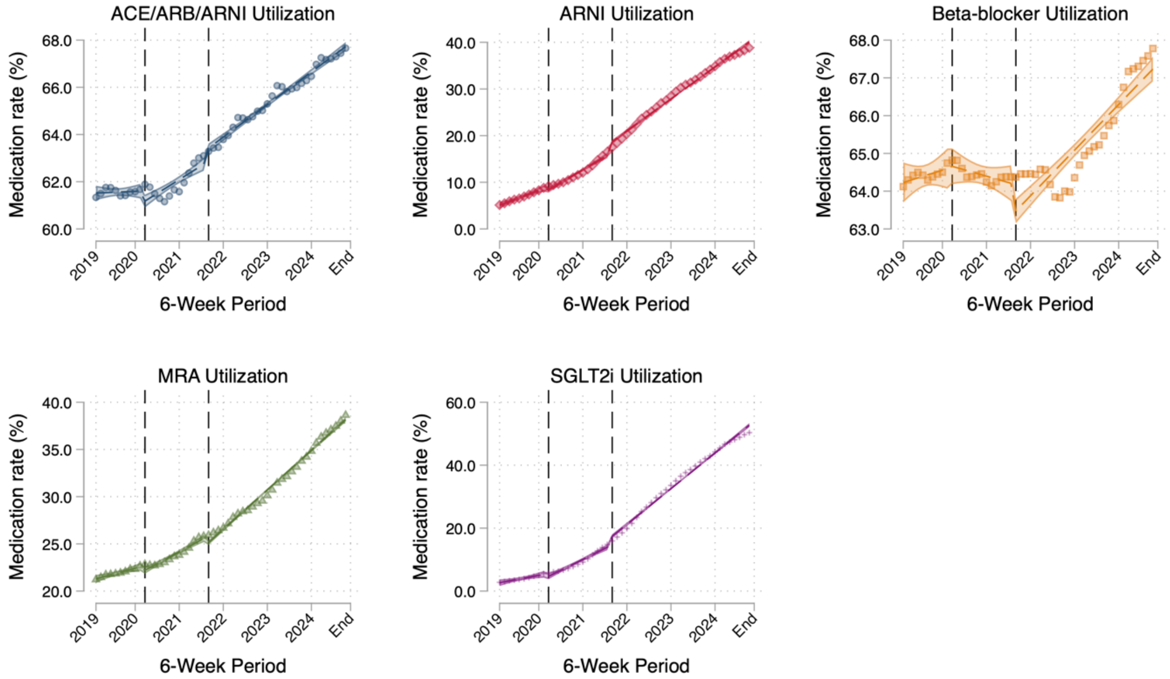

ACEI: angiotensin-converting enzyme inhibitor. ARB: angiotensin receptor blocker. ARNI: angiotensin receptor-neprilysin inhibitor. MRA: mineralocorticoid receptor antagonist. SGLT2i: sodium glucose cotransporter 2 inhibitor.

First vertical line indicates transition from pre-COVID to early COVID phase (March 2020). Second vertical line indicates transition from early COVID to late COVID phase (August 2021).

medRxiv preprint doi: https://doi.org/10.1101/2025.10.13.25337954; this version posted October 15, 2025. The copyright holder for this preprint (which was not certified by peer review) is the author/funder, who has granted medRxiv a license to display the preprint in perpetuity.
It is made available under a CC-BY 4.0 International license .

Figure S4: Seasonal Effects on Primary Care and Cardiology Visits

Figure S4A: Primary Care Seasonal Patterns by Visit Type

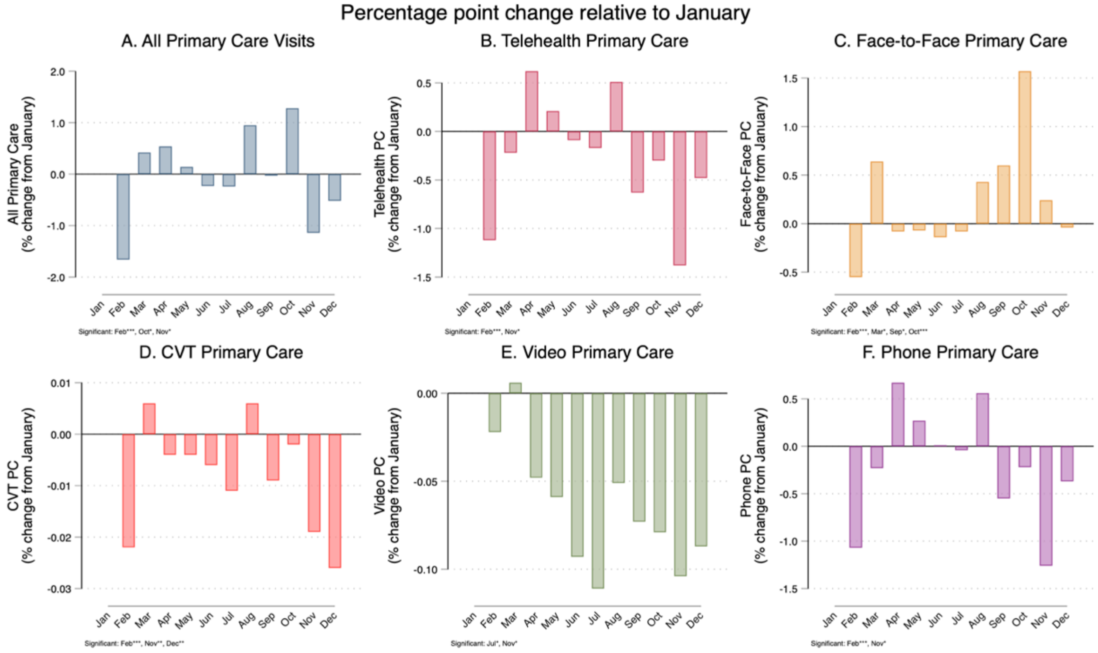

* p<0.05, ** p<0.01, *** p<0.001

Figure S4B: Cardiology Seasonal Patterns by Visit Type

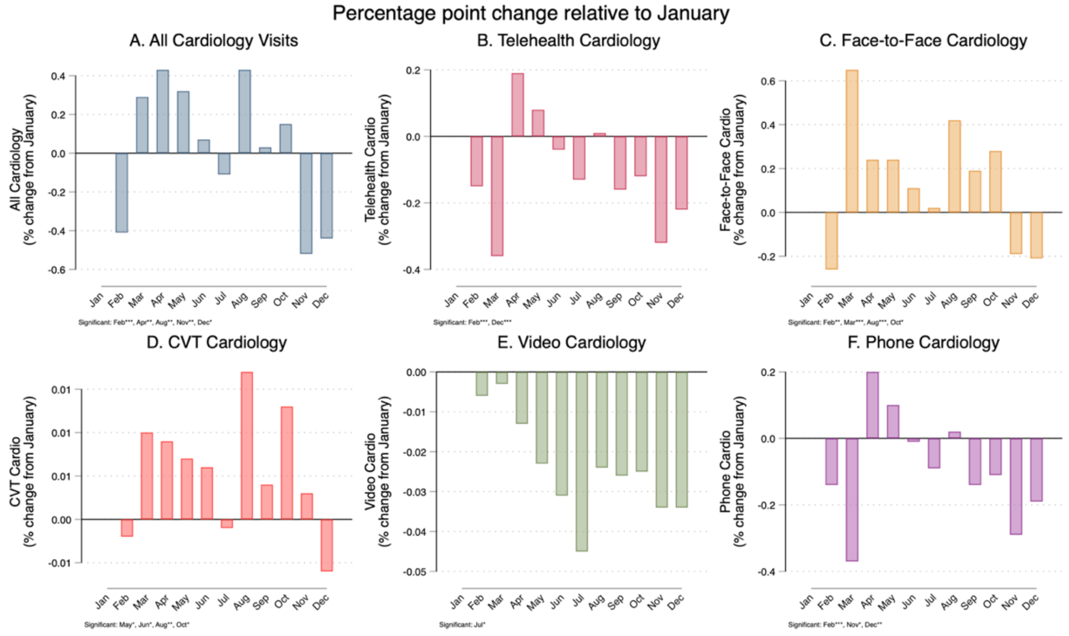

* p<0.05, ** p<0.01, *** p<0.001

medRxiv preprint doi: https://doi.org/10.1101/2025.10.13.25337954; this version posted October 15, 2025. The copyright holder for this preprint (which was not certified by peer review) is the author/funder, who has granted medRxiv a license to display the preprint in perpetuity.
It is made available under a CC-BY 4.0 International license .

Figure S5: Individual Hospitalization and Mortality Trends Between 2019 and 2024

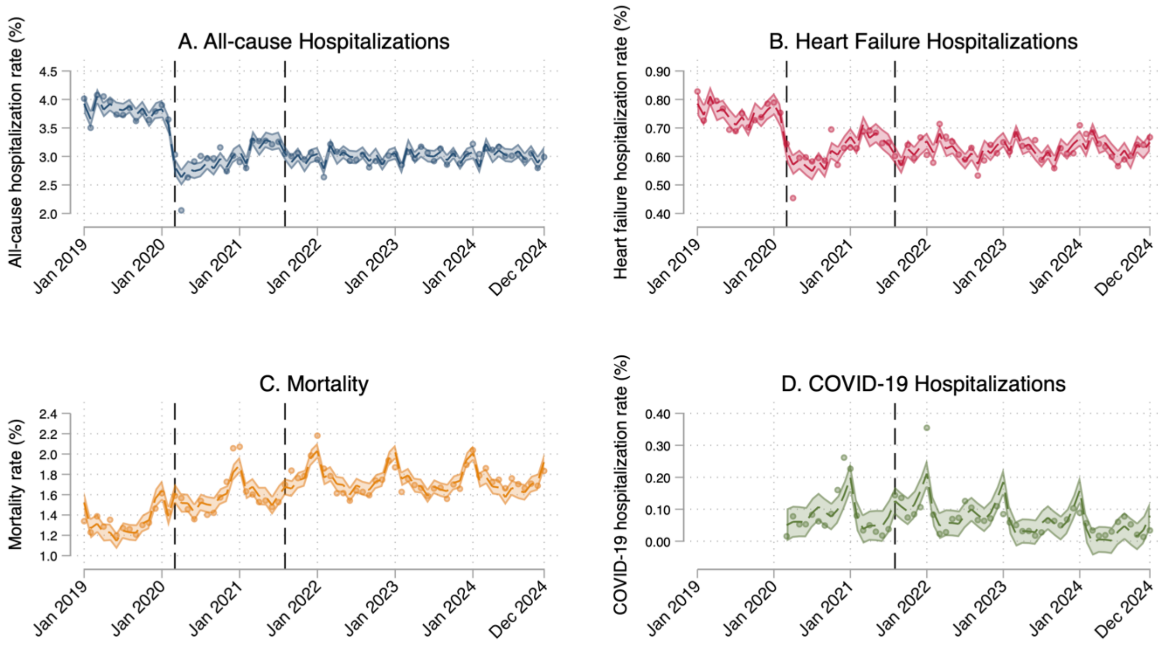

First vertical line indicates transition from pre-COVID to early COVID phase (March 2020). Second vertical line indicates transition from early COVID to late COVID phase (August 2021).

Shaded areas represent 95% confidence intervals.

medRxiv preprint doi: https://doi.org/10.1101/2025.10.13.25337954; this version posted October 15, 2025. The copyright holder for this preprint (which was not certified by peer review) is the author/funder, who has granted medRxiv a license to display the preprint in perpetuity.
It is made available under a CC-BY 4.0 International license .

Figure S6: Seasonal Effects on Hospitalization and Mortality

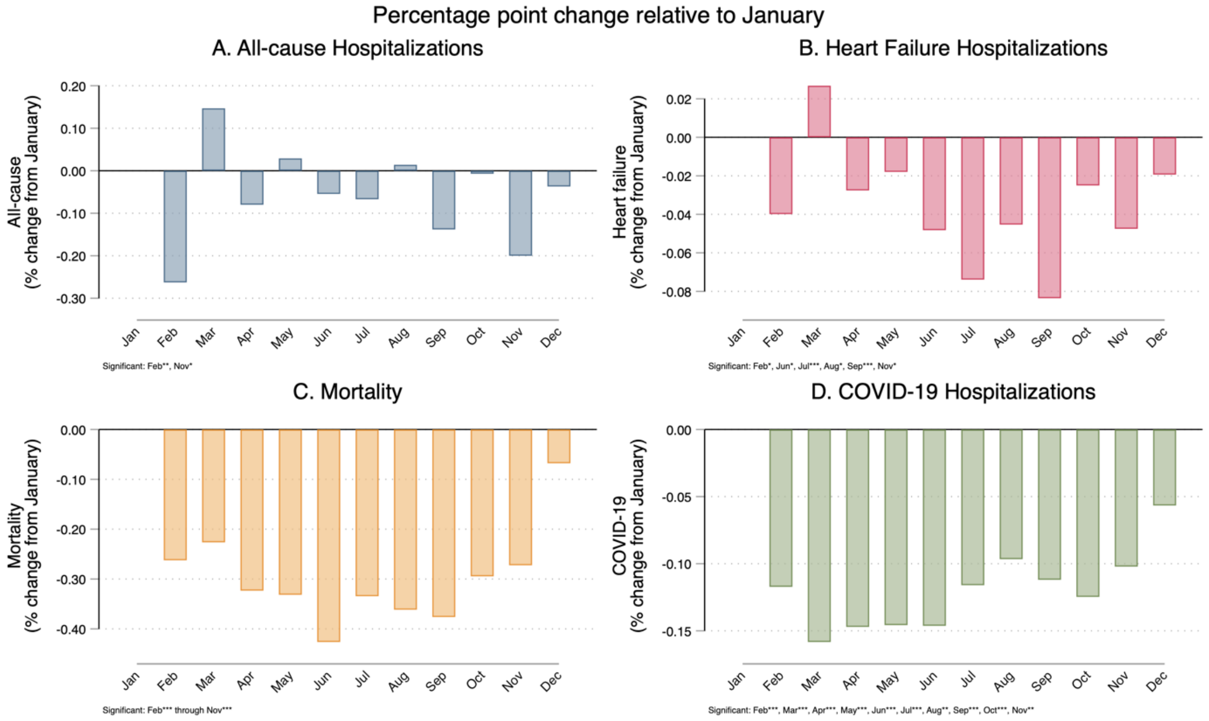

* p<0.05, ** p<0.01, *** p<0.001

medRxiv preprint doi: https://doi.org/10.1101/2025.10.13.25337954; this version posted October 15, 2025. The copyright holder for this preprint (which was not certified by peer review) is the author/funder, who has granted medRxiv a license to display the preprint in perpetuity.
It is made available under a CC-BY 4.0 International license .

Figure S7: Kaplan Meier Curves for Time-To-Event Outcomes

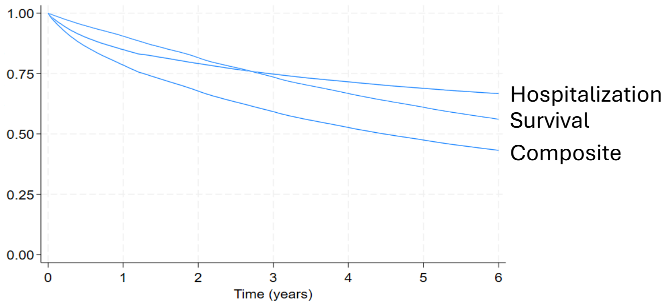

Time (years)	0	1	2	3	4	5	6
**Hospitalization -free**	210,535	165,421	142,722	128,000	110,000	100,000	90,999
**Overall Survival**	210,535	190,606	171,812	154,000	135,000	125,000	118,114
**Composite Endpoint-free**	210,535	165,421	142,722	125,000	108,000	98,000	90,999
